# Characterisation of oral and i.v. glucose handling in truncally vagotomised subjects with pyloroplasty

**DOI:** 10.1530/EJE-13-0264

**Published:** 2013-08

**Authors:** Astrid Plamboeck, Simon Veedfald, Carolyn F Deacon, Bolette Hartmann, André Wettergren, Lars B Svendsen, Søren Meisner, Claus Hovendal, Filip K Knop, Tina Vilsbøll, Jens J Holst

**Affiliations:** 1 Diabetes Research Division, Department of Internal MedicineGentofte Hospital, University of CopenhagenHellerupDenmark; 2 The Novo Nordisk Foundation Center for Basic Metabolic ResearchDepartment of Biomedical Sciences Panum Institute, University of CopenhagenCopenhagenDenmark; 3 Department of Surgical Gastroenterology and Liver TransplantationRigshospitalet, University of CopenhagenCopenhagenDenmark; 4 Department of Surgical GastroenterologyBispebjerg Hospital, University of CopenhagenCopenhagenDenmark; 5 Department of Surgical GastroenterologyOdense University Hospital, University of OdenseOdenseDenmark

## Abstract

**Objective:**

Glucagon-like peptide 1 (GLP1) is rapidly inactivated by dipeptidyl peptidase 4 (DPP4), but may interact with vagal neurons at its site of secretion. We investigated the role of vagal innervation for handling of oral and i.v. glucose.

**Design and methods:**

Truncally vagotomised subjects (*n*=16) and matched controls (*n*=10) underwent 50 g-oral glucose tolerance test (OGTT)±vildagliptin, a DPP4 inhibitor (DPP4i) and isoglycaemic i.v. glucose infusion (IIGI), copying the OGTT without DPP4i.

**Results:**

Isoglycaemia was obtained with 25±2 g glucose in vagotomised subjects and 18±2 g in controls (*P*<0.03); thus, gastrointestinal-mediated glucose disposal (GIGD) – a measure of glucose handling (100%×(glucose_OGTT_−glucose_IIGI_/glucose_OGTT_)) – was reduced in the vagotomised compared with the control group. Peak intact GLP1 concentrations were higher in the vagotomised group. Gastric emptying was faster in vagotomised subjects after OGTT and was unaffected by DPP4i. The early glucose-dependent insulinotropic polypeptide response was higher in vagotomised subjects. Despite this, the incretin effect was equal in both groups. DPP4i enhanced insulin secretion in controls, but had no effect in the vagotomised subjects. Controls suppressed glucagon concentrations similarly, irrespective of the route of glucose administration, whereas vagotomised subjects showed suppression only during IIGI and exhibited hyperglucagonaemia following OGTT. DPP4i further suppressed glucagon secretion in controls and tended to normalise glucagon responses in vagotomised subjects.

**Conclusions:**

GIGD is diminished, but the incretin effect is unaffected in vagotomised subjects despite higher GLP1 levels. This, together with the small effect of DPP4i, is compatible with the notion that part of the physiological effects of GLP1 involves vagal transmission.

## Introduction

Glucagon-like peptide 1 (GLP1), released from intestinal L cells, acts as an important signalling peptide with regards to glucose disposal, gastrointestinal motility and satiety (1), and incretin-based therapies are now widely used in the treatment of type 2 diabetes (2, 3). While the clinical benefits of GLP1-based therapies are relatively clear, the physiological mechanisms by which endogenous GLP1 exerts its effects remain obscure. The peripheral concentrations of intact GLP1 are very low because the native peptide is degraded rapidly by dipeptidyl peptidase 4 (DPP4) (4, 5, 6, 7), suggesting that the many effects of GLP1 may be mediated via non-endocrine signalling. Several lines of evidence suggest that GLP1 could act locally before being degraded (8, 9, 10, 11, 12): i) nodose ganglion cells express the GLP1 receptor (GLP1R) gene (13); ii) vagal nerve terminals in the portal vein contain GLP1Rs (14); and iii) GLP1 infusions in rats increase impulse generation in vagal afferent fibres (8, 9). It is therefore plausible that vagal sensory afferents in the lamina propria and/or portal vein mediate GLP1 actions (15).

In animal models, subdiaphragmatic vagal deafferentation, which ablates all vagal afferents but leaves ∼50% of the efferent nerves intact, or vagal sensory ablation using capsaicin, can be used to elucidate the role of vagal afferent fibres (10, 11, 16), but such interventions are obviously not possible in humans. One possibility is to examine subjects with a truncal vagotomy. Observations dating from the 1970s indicated that truncally vagotomised duodenal ulcer patients have significantly reduced insulin responses during oral but not i.v. glucose tolerance tests, despite earlier and greater postprandial glucose responses compared with non-operated controls (17, 18). Furthermore, patients with gastric or oesophageal cancer, treated with reconstructive surgery and vagotomy, had glucose intolerance associated with hyperglucagonaemia and insulinopenia, and it was concluded that loss of vagal signalling might explain the observed impairment in first-phase insulin secretion (19). These observations were made before the discovery of GLP1, but today it is assumed that a loss of GLP1 signalling via vagal afferents could, at least in part, explain such derangements in both insulin and glucagon secretion.

This study aimed to investigate the role of vagal innervation for handling of oral and i.v. glucose and the associated changes in the secretion of the incretin hormones, including the gastrointestinal-mediated glucose disposal (GIGD) – a measure of glucose handling – which includes not only the incretin effect but also other factors, including the impact of the glucagonostatic effects of GLP1 and neural reflexes (20, 21). Subjects with truncal vagotomy were investigated during oral glucose tolerance test (OGTT) with and without DPP4 inhibition and isoglycaemic i.v. glucose infusion (IIGI), copying the plasma glucose (PG) during the OGTT (without DPP4 inhibition). Matched healthy subjects with intact vagal innervation were studied for comparison.

## Materials and methods

### Study protocol

Oral and written informed consent was obtained from all participants. The study was conducted in accordance with the Helsinki II declaration, approved by the Scientific Ethical Committee of the Capital Region of Denmark (registration no. H-A-2009-060) and by the Danish Data Protection Agency (journal no. 2009-41-3740), and was registered at www.clinicaltrials.gov (ClinicalTrials.gov ID: NCT01176760).

### Subjects

Inclusion criteria included informed consent, normal haemoglobin and, for the vagotomised group, a truncal vagotomy. Exclusion criteria included obesity (BMI above 30 kg/m^2^), diabetes, inflammatory bowel disease, gut resection (except appendectomy), kidney or liver disease (S-kreatinin >150 μM and/or albuminuria, alanine and/or aspartate amino transferases above two times normal plasma values) and medication that could not be paused for 12 h.

Seven subjects who had undergone surgery (truncal vagotomy and pyloroplasty ad modum Heineke-Mikulic) for uncomplicated duodenal ulcer were included. In addition, nine subjects, treated in the period from 2002 until 2008, with cardia resection ad modum Ivor Lewis, including truncal vagotomy and pyloroplasty for oesophageal cancer were included. However, in order to avoid a delayed or incomplete gastric emptying, a pyloroplasty is also performed in connection with the vagotomy, leading to an altered pattern of gastric emptying (22, 23). All subjects treated with cardia resection were postoperatively treated with chemo and radiation therapy and none showed any sign of tumour recurrence or disseminated disease. Examination of the results from the experiments performed in this study revealed no differences between the duodenal ulcer and oesophageal cancer patients (Supplementary Tables 1–5, see section on supplementary data given at the end of this article). Therefore, the data were pooled and analysed together. All subjects operated for oesophageal cancer were treated with proton pump inhibitors (for prevention of gastro-oesophageal reflux). Other medications in the vagotomised group included statins (dyslipidaemia, *n*=3), anticoagulant drugs (dipyramidol, clopidogrel and/or acetylic salicylic acid, *n*=3), antihypertensive drugs (angiotensin-converting enzyme (ACE) inhibitors, calcium antagonists and/or β-blockers, *n*=5) or inhalation therapy for asthma (β-agonist and glucocorticoids, *n*=1). The vagotomies in the duodenal ulcer subjects were considered complete, as evidenced by >90% reduction in insulin-induced peak acid output at 3 months and 5 years after surgery. Moreover, no participants had a history of recurrent ulcer disease, taken to indicate a lack of re-innervation. No such data were available for the oesophageal cancer subjects. However, their operation (cardia resection ad modum Ivor Lewis) definitely involves truncal vagotomy due to the anatomical proximity of the cancer to the vagus nerve and therefore also included a pyloroplasty (24).

Ten healthy, matched subjects without family history of diabetes served as controls. Medications included antihypertensive drugs (ACE inhibitors, calcium antagonists and/or thiazides, *n*=3), anticoagulant therapy (acetylic salicylic acid, *n*=3), therapy for benign prostate hypertrophy (α-blocker, *n*=1) or analgesics for lumbago (opioid agonist, *n*=1). All medications were paused at least 12 h before examinations. Subject characteristics are shown in Table 1. Overall, participants had no clinically relevant changes in biochemical parameters.

### Experimental protocol

Subjects were studied in the fasting state (12-h fast) on four occasions, each separated by more than 24 h. All were instructed to maintain an unchanged lifestyle but to abstain from alcohol and exercise in the 24 h leading up to examination. Smoking was prohibited for the last 12 h before each experimental day. Subjects were seated and a cannula was inserted into a cubital vein for collection of arterialised blood samples (cannulated forearm was placed in a heating box (50 °C) throughout each experiment).

Participants underwent a 15-min chew-and-spit ‘sham-feeding’ with a standardised test meal consisting of a breakfast platter (including eggs, bacon, bread, butter, cheese, marmalade, yoghurt, fruits, pancakes, orange juice and tea or coffee) in order to evaluate vagal integrity (25). Participants were observed closely during the test and were specifically instructed to avoid swallowing any food, drink or saliva. Blood was drawn at −15, 0, 15, 30, 45 and 60 min, and distributed into chilled tubes containing EDTA for the analysis of total GLP1 and pancreatic polypeptide (PP). For bedside PG measurements, blood was collected in fluoride tubes and centrifuged immediately (7400 ***g***; 1 min at room temperature).

On two separate occasions, subjects underwent an OGTT either with or without DPP4 inhibition in randomised order. Twelve hours and one hour before the OGTT with DPP4 inhibition, the subjects ingested 50 mg vildagliptin (Galvus, Novartis Europharm Ltd.). During the OGTTs, the subjects ingested 50 g glucose dissolved in 200 ml water containing 1.5 g paracetamol (Panodil, GlaxoSmithKline A/S) at an even rate over 15 min. Paracetamol was added to the oral glucose for the evaluation of gastric emptying. Manifestation of paracetamol in the blood can be used as an indirect measurement of gastric emptying (of liquids), as it is poorly absorbed from the stomach but rapidly so from the proximal part of the small intestine (26). Arterialised blood was drawn at 15, 10 and 0 min before and 5, 10, 15, 20, 25, 30, 35, 40, 45, 50, 55, 60, 75, 90, 120, 180 and 240 min after glucose ingestion, and distributed into chilled tubes containing EDTA plus aprotinin (500 kIU/ml blood; Trasylol; Bayer Corp.) and DPP4 inhibitor (DPP4i; valine–pyrrolidide, 0.01 mM final concentration, a gift from Novo Nordisk, Bagsværd, Denmark) for analyses of glucagon, intact and total GLP1 and intact glucose-dependent insulinotropic polypeptide (GIP). Blood for insulin and C-peptide analyses was collected in dry tubes for coagulation (20 min at room temperature), and into lithium–heparin tubes for paracetamol measurement. After centrifugation (1200 ***g*** for 20 min at 4 °C), plasma for GLP1, GIP, glucagon and paracetamol analyses was stored at −20 °C, and serum for insulin and C-peptide analyses was stored at −80 °C. For bedside PG measurements, blood was collected into fluoride tubes and centrifuged immediately (7400 ***g*** for 1 min at room temperature).

On the IIGI day, cannulas were inserted into antecubital veins in both arms; one for blood sample collection (as described above) and one for glucose infusion. Sterile 20% (wt/vol) glucose was infused at a variable rate, aiming to duplicate the PG profile from the OGTT day without DPP4 inhibition. Blood was sampled as described above.

Before each experiment, the urinary bladder was emptied, and total urine production during each experiment was collected for measurement to exclude renal glucose excretion. This procedure was performed for the first four subjects of both groups, but as no urinary glucose was detected in any of these participants, this procedure was not continued (data not shown).

### Laboratory analyses

PG concentrations were measured by the glucose oxidase method (Yellow Springs Instrument model 2300 STAT plus analyzer; YSI, Inc., Yellow Springs, OH, USA). Serum insulin and C-peptide concentrations were measured using two-sided electrochemiluminescence immunoassays (Roche/Hitachi Modular Analytics; Roche Diagnostic GmbH). Plasma samples for PP, GLP1, GIP and glucagon measurements were extracted with 70% ethanol (final concentrations) before analysis by RIA. PP was measured using a mid-region specific antibody, code no HYB 347-07 (Statens Serum Institut, Copenhagen, Denmark). Total GLP1 was assayed using antiserum 89390, which has an absolute requirement for the intact amidated C-terminus of the molecule, while intact GLP1 was measured using a two-site (sandwich) ELISA. Intact GIP was measured using N-terminally directed antisera code nos 98171. Glucagon immunoreactivity was determined using the C-terminally directed antiserum 4305, which measures glucagon of pancreatic origin. Sensitivities were below 2 pM and intraassay coefficients of variation better than 6% (27, 28, 29). Plasma paracetamol was measured by a routine enzymatic colorimetric assay (Ortho-Clinical Diagnostics, Johnson & Johnson, Birkerød, Denmark) for the Vitros 5.1. FS analyzer (30, 31).

### Calculations and statistical analyses

Results are reported as means±s.e.m.; a two-sided *P* value of <0.05 was taken to indicate significant difference. Statistical analyses were carried out using GraphPad Prism version 5.00 for Windows (GraphPad Software, San Diego, CA, USA). The data was tested using D'Agostino–Pearson omnibus K2 normality test for normal distribution. Two-way repeated-measures ANOVA and Bonferroni post-hoc tests were applied to test for differences in repeatedly measured values between days (i.e. absolute PG, hormone and paracetamol concentrations). For paired comparisons between single values (e.g. between baseline and area under the curve (AUC) values, incretin effect and GIGD), we used paired *t*-tests within the groups and unpaired *t*-tests for comparisons between groups in data that followed a normal distribution. Data that did not follow a normal distribution were tested using Wilcoxon's test for paired difference and Mann–Whitney *U* test for unpaired difference. Insulin resistance (IR) was calculated using the homeostatic model assessment of IR (HOMA–IR) (32). GIGD, which describes the impact of gastrointestinal factors on glucose disposal following OGTT compared with IIGI, was calculated using the formula: GIGD (%)=100%×(glucose_OGTT_−glucose_IIGI_)/glucose_OGTT_ (21). AUC and incremental AUC (iAUC; i.e. baseline levels subtracted) were calculated using the trapezoidal rule. The incretin effect was calculated from the β cell secretory responses to oral and isoglycaemic i.v. glucose as follows: 100%×(AUC_OGTT_−AUC_i.v._)/AUC_OGTT_. Prehepatic insulin secretion rates (ISRs) were calculated by deconvolution of peripheral C-peptide concentrations and application of population-based parameters for C-peptide kinetics, using the ISEC Software (33, 34). To evaluate β cell glucose sensitivity (βGS; a measure of the dose–response relationship between glucose concentration and ISR), the time when peak glucose concentration was reached for each subject on each experimental day was identified, and ISR values from time point 0 min to the time for peak glucose were plotted against the corresponding PG concentrations. The slopes of these linear relationships reflect changes in ISR per mM increase in PG (35). The insulinogenic index (IGI) was calculated using the following formula: (insulin_30 min_−insulin_fasting_)/(glucose_30 min_−glucose_fasting_). To adjust for differences in insulin sensitivity, individual βGSs were related to HOMA2–IR by calculating the disposition index (DI) as DI_β__GS_ (βGS×HOMA2–IR^−1^) and DI_IGI_ (IGI×HOMA2–IR^−1^). The absolute difference between responses to the OGTT with and without DPP4i (the effect of DPP4 inhibition) was calculated from total AUC (tAUC) for ISR and from iAUC for PG and gastrointestinal hormones using the following formula: iAUC_OGTT__+__DPP4_−iAUC_OGTT_.

## Results

### Sham-feeding

**Figure 1:**
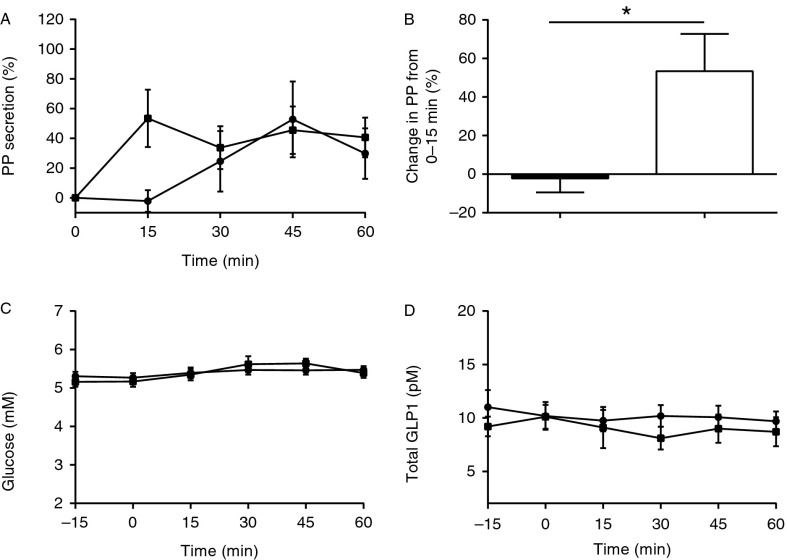
(A) Time course of changes in pancreatic polypeptide (PP) secretion before and after ‘sham-feeding’ in vagotomised (circles) and control (squares) subjects. (B) Changes in PP secretion immediately after ‘sham-feeding’ in vagotomised (black bar) and control (white bar) subjects. (C) Plasma glucose before and after ‘sham-feeding’ in vagotomised (circles) and control (squares) subjects. (D) Total GLP1 before and after ‘sham-feeding’ in vagotomised (circles) and control (squares) subjects. Data are shown as means±s.e.m. *Significantly reduced PP secretion in the first 15 min after ‘sham-feeding’ in the vagotomised subjects (*P*<0.03).

Vagotomised and control subjects had similar baseline PP values (23±6 vs 26±6 pM, *P*=NS; Fig. 1A). PP levels increased in 11 of the 16 (69%) vagotomised and 8 of the 10 (80%) control subjects during the 1 h after sham-feeding, but the time-to-peak was delayed in the vagotomised group (36±4 vs 19±4 min, *P*<0.02). As judged by the PP rise during the first 15 min (cephalic phase), no vagal stimulation occurred in the vagotomised subjects (percentage increase in PP secretion after ‘sham-feeding’: −2±7 vs 53±20%, *P*<0.03 respectively; Fig. 1B).

Baseline PG values were similar in vagotomised and control subjects (5.3±0.1 vs 5.2±0.1 mM, *P*=NS), and were followed by a very small rise in PG in both groups (*P*<0.05) (Fig. 1C). Baseline GLP1 levels were similar in both groups (10.2±1.4 vs 10.0±0.9 pM, *P*=NS) and were unchanged by ‘sham-feeding’ (Fig. 1D).

### OGTT and IIGI

#### Paracetamol

**Figure 2:**
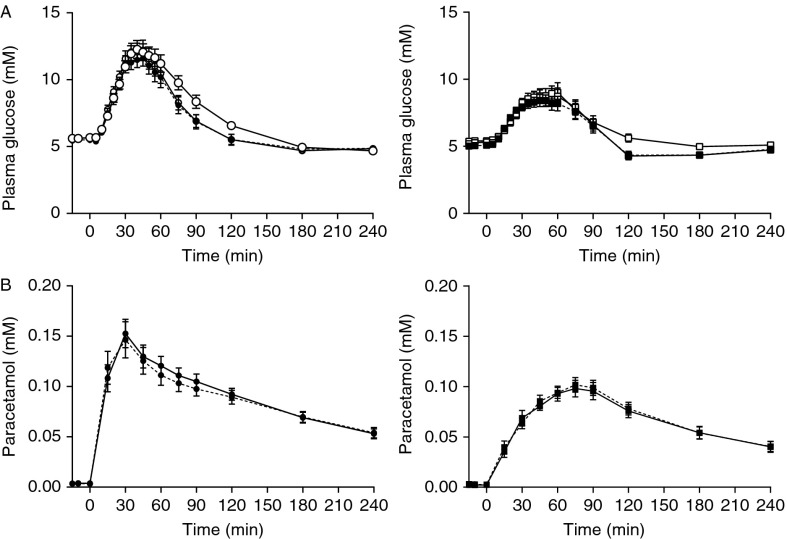
Time course of plasma glucose (A) during 50 g-oral glucose tolerance test (OGTT) (filled symbols) with (broken line) and without (solid line) DPP4 inhibition and isoglycaemic i.v. glucose infusion (IIGI) (open symbols) and paracetamol (B) during 50 g-OGTT (filled symbols) with (broken line) and without (solid line) DPP4 inhibition in vagotomised (left panel, circle) and control (right panel, square) subjects. Data are shown as means±s.e.m.

Gastric emptying was faster in vagotomised compared with control subjects (time-to-peak paracetamol: 34±5 vs 77±8 min respectively, *P*<0.0001; Fig. 2B).

#### Glucose and GIGD

Fasting PG was similar in both groups on the two experimental days (Fig. 2A, Table 2), but peak PG concentrations were higher in the vagotomised subjects (13.2±0.6 vs 9.2±0.5, *P*<0.0001). However, all participants exhibited normal 2-h PG values (vagotomised, 5.5±0.4 mM; controls, 4.3±0.3 mM). Isoglycaemia, evaluated by two-way repeated-measures ANOVA (PG during OGTT vs PG during adjustable i.v. glucose infusion), followed by Bonferroni post-hoc tests, was achieved (*P*=NS), although the AUC_PG_ values were somewhat larger during IIGIs compared with corresponding OGTTs in both groups (Table 2). However the difference between the AUCs during OGTT and IIGI was exactly the same in both groups (10.7±2.9 vs controls 7.9±1.5%, *P*=NS). During the first 75 min, however, there was complete isoglycaemia as evaluated by both two-way repeated-measures ANOVA and AUC in both vagotomised and control subjects (Table 2). Therefore, the incretin effect and AUCs for all the hormone responses were also calculated for this period (Tables 3 and 4). Isoglycaemia required 25±2 g glucose in vagotomised subjects and 18±2 g in controls (*P*<0.03), resulting in a significantly reduced GIGD in the vagotomised group (49±4 vs 63±4%, *P*<0.03). The glucose infusions were stopped after 54±4 min in vagotomised subjects and at 65±3 min in controls (*P*=NS).

#### GLP1

**Figure 3:**
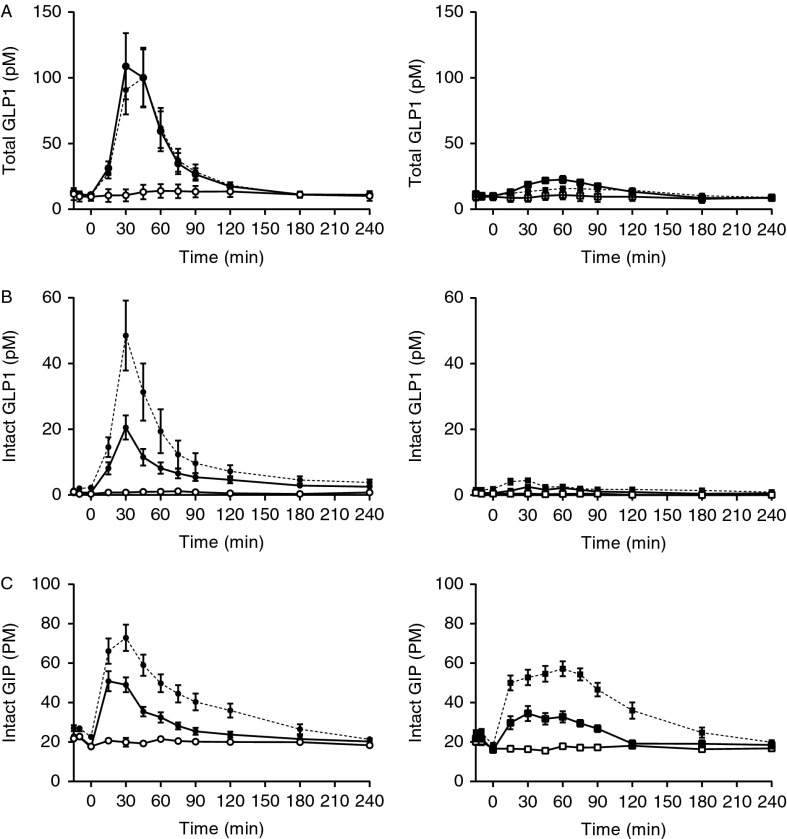
Time course of total GLP1 (A), intact GLP1 (B) and intact GIP (C) during 50 g-oral glucose tolerance test (OGTT) (filled symbols) with (broken line) and without (solid line) DPP4 inhibition and isoglycaemic i.v. glucose infusion (IIGI) (open symbols) in vagotomised (left panel, circles) and control (right panel, squares) subjects. Data are shown as means±s.e.m.

Fasting total and intact GLP1 levels were similar in both groups. However, vagotomised subjects had greater than fivefold higher peak levels (total GLP1: 121±29 vs 24±3 pM, *P*<0.02 (Fig. 3A); intact GLP1: 22±3 vs 3±2 pM, *P*<0.0003 (Fig. 3B)) and AUC (Tables 2 and 3) during oral glucose compared with controls. GLP1 levels were unchanged during IIGI in both groups.

#### Intact GIP

Fasting intact GIP concentrations were similar between the groups, and as expected, GIP levels increased significantly during the OGTT, but not during IIGI (Fig. 3D, Table 2). The two groups had similar responses (AUCs) to oral glucose during the whole experiment (Table 2), although peak GIP values (54±4 vs 36±4 pM, *P*<0.007) and AUCs during the first 75 min (Table 3) were significantly higher in the vagotomised subjects.

#### Insulin, C-peptide, ISR and incretin effect

**Figure 4:**
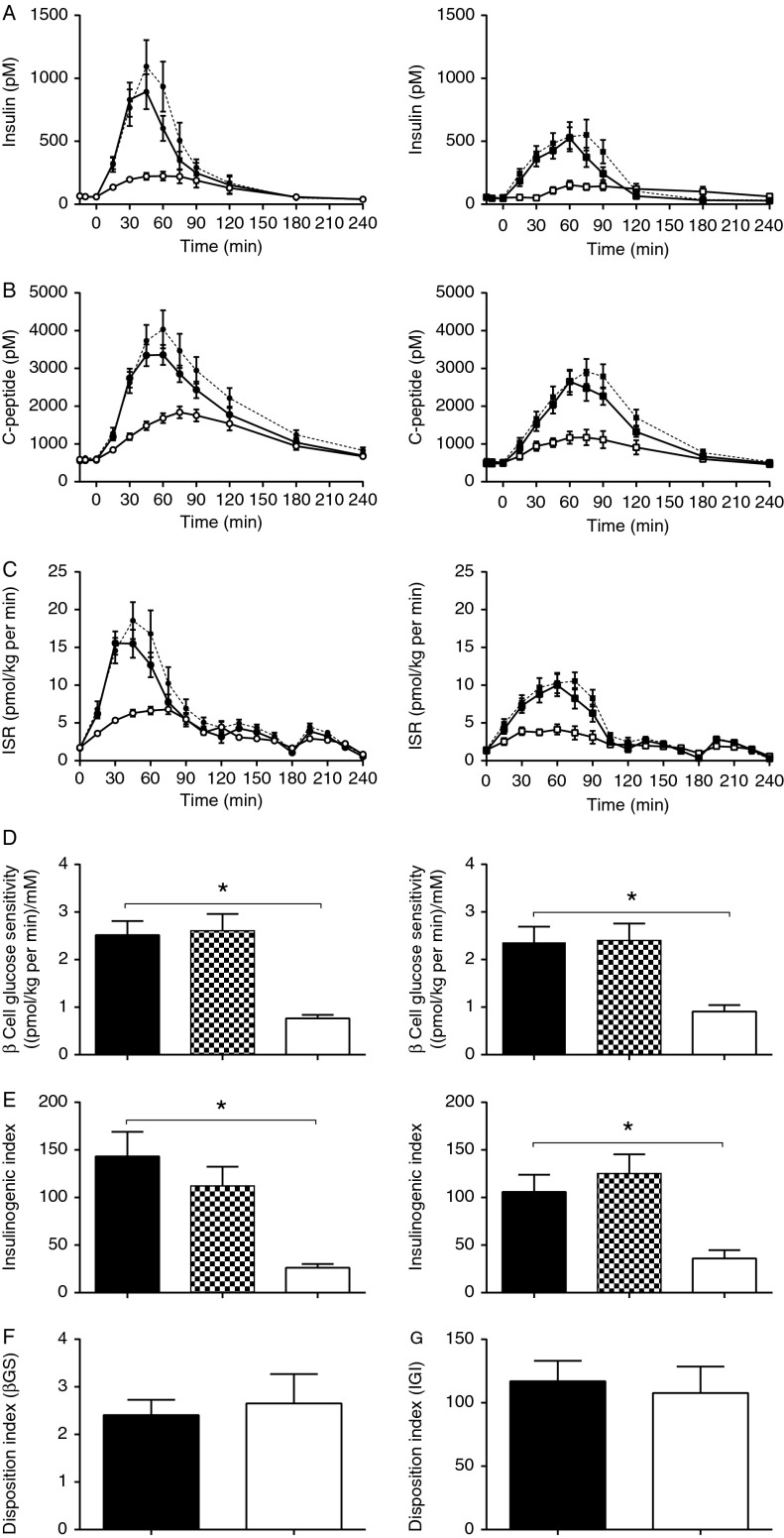
Time course of insulin (A), C-peptide (B) and ISR (C) during 50 g-oral glucose tolerance test (OGTT) (filled symbols) with (broken line) and without (solid line) DPP4 inhibition and isoglycaemic i.v. glucose infusion (IIGI) (open symbols) in vagotomised (left panel, circles) and control (right panel, squares) subjects and β cell glucose sensitivity (βGS) (D), insulinogenic index (IGI; E) during 50 g-OGTT with (checked bars) and without (black bars) DPP4 inhibition and IIGI (white bars) in vagotomised (left panel) and control (right panel) subjects, disposition index (DI) using βGS (F) and DI using IGI (G) during 50 g-OGTT in vagotomised (black) and control (white) subjects. Data are shown as means±s.e.m. *Significantly reduced βGS and IGI after i.v. compared with oral glucose load in both vagotomised and control subjects (*P*<0.002).

The two groups had similar IR (Table 1). Insulin, C-peptide and ISR responses were significantly enhanced in response to oral compared with i.v. glucose in both groups, but vagotomised subjects demonstrated a greater β cell response, especially during the OGTT (Fig. 4A, B and C, Tables 2, 3 and 4). However, the incretin effect calculated from the time period with absolute isoglycaemia (insulin, C-peptide or ISR AUCs) did not differ between the groups. When calculated from C-peptide iAUCs and tAUCs during the whole experiment, the incretin effect was reduced in vagotomised subjects (Table 4).

#### Measures of β cell function

β Cell responsiveness to changes in PG (βGS and IGI) was significantly higher during oral vs i.v. glucose in both groups. Notably, there were no differences between the groups on either day. Furthermore, β cell function (derived from either βGS or IGI), corrected for insulin sensitivity and expressed as the DI, was also similar (Fig. 4D, E, F and G).

#### Glucagon

**Figure 5:**
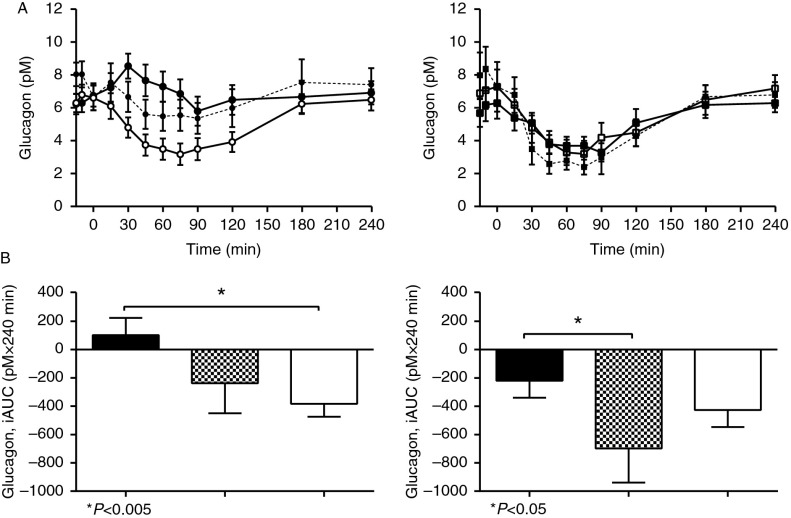
(A) Time course of glucagon during 50 g-oral glucose tolerance test (OGTT) (filled symbols) with (broken line) and without (solid line) DPP4 inhibition and isoglycaemic i.v. glucose infusion (IIGI) (open symbols) in vagotomised (left panel, circles) and control (right panel, squares) subjects. (B) Incremental area under the curve (AUC) values for glucagon during 50 g-OGTT with (checked bars) and without (black bars) DPP4 inhibition and IIGI (white bars) in vagotomised (left panel) and control (right panel) subjects. Data are shown as means±s.e.m. *Significantly reduced glucagon secretion after i.v. compared with oral glucose load in vagotomised subjects (*P*<0.005, left panel) and *significantly reduced glucagon secretion during DPP4 inhibition in control subjects (*P*<0.05, right panel).

Fasting concentrations of glucagon were comparable (Fig. 5A and B, Table 2), but vagotomised subjects exhibited different responses to the two glucose stimuli. Glucagon levels were suppressed during IIGI, but increased following OGTT (*P*<0.0001; post-hoc analysis showing significant differences at 30, 45, 60 and 75 min; Fig. 5A and B, Tables 2 and 3).

### OGTT with and without DPP4 inhibition

#### Paracetamol

Gastric emptying was unaffected by DPP4 inhibition in both the vagotomised (time-to-peak paracetamol: 34±5 (OGTT) vs 39±8 min (OGTT+DPP4 inhibition), *P*=NS) and control (time-to-peak acetaminophen: 77±8 (OGTT) vs 80±2 min (OGTT+DPP4 inhibition), *P*=NS) subjects (Fig. 2B, Table 5).

#### Glucose

DPP4 inhibition reduced fasting PG in both the vagotomised and control subjects (Fig. 2A, Table 6). However, the DPP4 inhibition did not affect the overall glucose profile in either group evaluated by iAUC and two-way repeated measurements ANOVA with Bonferroni post-hoc tests (*P*=NS).

#### GLP1

Fasting total GLP1 levels were similar and unaffected by DPP4 inhibition in both groups. In the vagotomised group, DPP4 inhibition increased fasting levels of intact GLP1 (Fig. 3A and B, Tables 5 and 6). Total GLP1 secretion was approximately fivefold higher in vagotomised subjects after OGTT compared with controls as shown earlier. DPP4 inhibition reduced both iAUC (Table 6) and peak levels of total GLP1 (24±3 vs 17±1 pM, *P*<0.02) in the control groups, but not in the vagotomised group, neither with respect to iAUC (Table 6) nor peak total GLP1 (121±29 vs 100±26 pM, *P*=NS). Intact GLP1 was, as expected, increased by DPP4 inhibition in both groups (peak: vagotomised: 22±3 vs 55±10 pM, *P*<0.0006; controls: 3±1 vs 5±1 pM, *P*<0.02).

#### Intact GIP

DPP4 inhibition did, as expected, increase both baseline levels (Fig. 3D, Table 6) and peak levels of intact GIP during OGTT (vagotomised: 54±4 vs 77±7 pM, *P*<0.0008; controls: 36±4 vs 57±4 pM, *P*<0.0001). The two groups also had similar and increased responses (iAUCs) to oral glucose during DPP4 inhibition. However, GIP levels were higher in the vagotomised subjects during the first 30 min after the OGTT with DPP4 inhibition (*P*<0.01; post-hoc analysis showing significant differences at 15 and 30 min; Tables 5 and 6).

#### Insulin, C-peptide and ISR

In the control subjects, DPP4 inhibition significantly enhanced insulin, C-peptide and ISR responses to the oral glucose load, whereas there was no significant effect in the vagotomised group (Fig. 4A, B and C, Table 6). There was no difference in the absolute effect or response to the DPP4i between the groups (Table 5).

#### Measures of β cell function

β Cell responsiveness to changes in PG (βGS and IGI) was unaffected by DPP4 inhibition and there were no differences between the groups on either days (Fig. 4D and E).

#### Glucagon

DPP4 inhibition did increase fasting glucagon levels in the control group (Fig. 5A, Table 6). DPP4 inhibition suppressed glucagon secretion in controls (Fig. 5B, Table 6). In contrast, vagotomised subjects were, as already mentioned, unable to suppress glucagon secretion following OGTT. However, during DPP4 inhibition there was a tendency towards a normalised glucagon response. The absolute effect of DPP4 inhibition (Table 5) was similar in the two groups.

## Discussion

The present findings indicate that vagotomy with pyloroplasty is associated with: i) accelerated gastric emptying of an oral glucose load; ii) altered glucose profile after OGTT and reduced GIGD; iii) more than fivefold higher OGTT-induced GLP1 responses and elevated initial GIP levels; iv) normal incretin effect; v) inappropriate glucagon responses after OGTT; and vi) small effects of DPP4 inhibition on PG, insulin and glucagon responses, despite high levels of both intact GLP1 and GIP.

The vagotomy procedure serves as the ‘intervention’ in this study, and it could be a concern that the vagotomies (particularly for the duodenal ulcer patients) were performed many years before the investigations. Therefore, in order to evaluate vagal integrity, all subjects underwent a chew-and-spit ‘sham-feeding’. Meal-induced PP secretion is characteristically biphasic, with the early cephalic phase being vagally mediated while the second prolonged phase is less dependent on vagal activity (36). An early PP response to sham-feeding can, therefore, be used as a reliable marker of vagal integrity, and was seen immediately in controls, but not in the vagotomised patients. Although it cannot be fully excluded that some vagal nerve regeneration may have occurred since the original operation, long-term studies in vagotomised rats reported no or only incomplete/partially disordered vagal regeneration (37, 38). However, given the complete lack of the early cephalic PP response in our patients, we believe that their vagotomies were still complete.

Accelerated gastric emptying, particularly for liquids, is a known consequence of the pyloroplasty performed in connection with vagotomy (22, 23) and, as expected, gastric emptying, evaluated using paracetamol manifestation in the blood, was faster in the vagotomised subjects compared with controls. This accelerated gastric emptying in the vagotomised subjects will lead to a more rapid delivery of nutrients to the small intestine, and will affect many of the parameters measured in this study, e.g. PG levels, GLP1 and GIP secretions, insulin and glucagon responses. While differences in gastric emptying might have been avoided if the oral glucose loads had been delivered directly into the duodenum, many of the participants were unwilling to have an intraduodenal tube. To minimise the emptying effects, the glucose load was reduced from the standard 75 to 50 g and ingested over 15 rather than 5 min. Gastric emptying was still faster in the vagotomised subjects, but none had any dumping symptoms.

Probably as a consequence of the accelerated gastric emptying in the vagotomised subjects with pyloroplasty, PG increased rapidly after oral glucose in these subjects, with peak concentrations reaching levels of diagnosis of diabetes (39). Nevertheless, PG returned to baseline within 2 h. Thus, vagotomised subjects with pyloroplasty have altered glucose profiles after OGTT with high peak values, but not impaired glucose tolerance *per se*, in accordance with current guidelines as judged by their 2 h PG values and previous observations in vagotomised subjects (17, 40). Isoglycaemia was achieved, judging from both ANOVA and AUCs, during the first 75 min of the PG profiles, but it turned out to be impossible to accurately match the glucose curves during the descending phase of the OGTT on the i.v. glucose day. Clearly, the subjects eliminated glucose very effectively after the OGTT, at a rate that exceeded maximum elimination during i.v. glucose. Consequently, the overall glucose excursions (AUC) were somewhat larger during the IIGIs and glucose-induced insulin responses are, therefore, also likely to be larger. Upon calculation of the incretin effect, this will lead to falsely low values while GIGD might also be underestimated. However, the difference in AUC during the OGTT and IIGI for the full PG profile was the same in both vagotomised and control subjects. It could, therefore, be argued that its impact on the hormone responses and further calculations might be the same in both groups. Nevertheless, we also calculated the incretin effect during the first 75 min (see below), where we had absolute isoglycaemia in both groups. The glucose infusions needed to obtain isoglycaemia were discontinued long before 75 min and therefore GIGD may be a reliable parameter in this study. The vagotomy and pyloroplasty were associated with a significantly reduced GIGD. GIGD describes the impact of gastrointestinal factors on glucose disposal following OGTT compared with IIGI, which is expressed as the difference between the amount of glucose ingested (and absorbed via the gastrointestinal tract) and that infused i.v. to mimic the PG curve after oral glucose. Therefore, GIGD describes not only the impact of the incretin effect, but rather includes all factors affecting postprandial PG concentrations (including, in addition to the incretin hormones, neural reflexes, activation of local afferent nerves, differences in glucagon secretion, first-pass hepatic glucose uptake, differences in portal and venous blood glucose concentrations, and any currently unknown factors) (21). Any of these could theoretically contribute to the reduced GIGD seen in the vagotomised subjects, including the neural reflexes and/or activation of local afferent nerves. However, in this study, gastric emptying should perhaps also be added to the list of factors influencing the GIGD. The higher glucose infusion rates needed in the vagotomised subjects in order to achieve comparable glucose profiles, and thereby the lower GIGD, could be a consequence of the higher peak glucose levels probably caused by the accelerated gastric emptying. DPP4 inhibition reduced fasting PG in both groups, but had no effect on the glucose excursions, similar to previous observations in healthy subjects (41).

The vagotomised subjects had more than fivefold higher GLP1 responses after oral glucose compared with controls. Truncal vagotomy leads to disturbances in gastrointestinal motility and secretion (42, 43), and both vagal afferents and efferents have been implicated in GLP1 secretion in rats (44). Based on this, lack of vagal signalling would be expected to reduce GLP1 secretion, but circulating GLP1 levels were actually substantially higher in the vagotomised patients. This rapid delivery of glucose distally in the gut, caused by the accelerated gastric emptying, could explain both the high peak PG and elevated GLP1 levels in these subjects; a pattern also seen after partial gastrectomy and in gastric by-pass-operated subjects (19, 45). These observations provide little support for the involvement of vagal pathways in stimulating GLP1 secretion in man. GIP tAUCs after oral or i.v. glucose were similar between the groups, but peak values after oral glucose were significantly higher in the vagotomised group, probably also reflecting accelerated gastric emptying. Regarding the effect of the DPP4i on the secretion of the incretin hormones, it was evident that GLP1 levels were significantly reduced in the control subjects and there was a similar tendency in the vagotomised subjects. This could reflect a feedback inhibition of GLP1 secretion after DPP4 inhibition, as previously shown (46). Nevertheless, both intact GLP1 and GIP levels were, as expected, increased after DPP4 inhibition in both groups.

β Cell function (as assessed from βGS, IGI and DI during i.v. glucose administration) was similar in both groups. Consistent with the high PG, GLP1 and GIP levels in the vagotomised patients after oral glucose, these subjects had larger insulin responses. However, despite this, the incretin effect, calculated in the conventional way for both groups for the first 75 min, where there was absolute isoglycaemia, was not increased. This in itself is remarkable considering the far higher levels of intact GLP1 and GIP after oral glucose in the vagotomised subjects, which might be expected to enhance β cell responsiveness directly. GIP and GLP1 are responsible for the incretin effect (47, 48, 49) and in healthy subjects they seem to contribute nearly equally (49), although one study indicated that GIP may contribute more to the incretin effect than GLP1 (50). No such studies have been performed in subjects with high postprandial levels of GLP1 (as observed in the vagotomised subjects in this study). However, gastrectomised patients have been shown to have elevated GLP1 levels after oral glucose compared with control subjects (51), whereas GIP levels were less affected (52). These subjects also have a rapid gastric emptying leading to hyperglycaemia followed by high insulin and hypoglycaemia (51). Gastric bypass is associated with postprandial hyperinsulinemia and increased GLP1 levels, suggesting that GLP1 in this situation contributes greatly to the changed postprandial glucose regulation and may be responsible for the reactive hypoglycaemia often seen in these patients. Moreover, in studies in healthy subjects, where similar levels of glucose and GLP1 were mimicked by i.v. infusions, insulin levels were elevated greatly and this resulted in pronounced reactive hypoglycaemia (53). A recent study using GLP1R blockade with exendin (9–39) showed that GLP1 contributes significantly to the hyperinsulinemia in the gastric bypass-operated subjects (54). Therefore, taken together, these findings are consistent with an impairment of the insulinotropic effect of GLP1 after vagotomy, as also shown in rodents (10, 11, 55). Furthermore, in contrast to GLP1, there are no experimental data suggesting that GIP effects are transmitted via vagal signalling (9, 13). The very high circulating levels of both intact GLP1 and GIP may therefore conceal any loss of vagal transmission of GLP1 effects. Supporting this view, sensory nerve denervation in mice abolished the insulinotropic effect of low, but not high, doses of GLP1 given i.v. (11). This was interpreted to suggest that neural reflexes are important for physiological (endogenous) GLP1 levels to stimulate insulin secretion, whereas at supra-physiological levels, the higher peripheral plasma concentrations may also directly activate the pancreatic β cell GLP1R. This may also explain why DPP4 inhibition had similar effects in vagotomised and control subjects. We hypothesised that activation of vagal sensory afferents by the higher splanchnic levels of active GLP1 (as opposed to systemic levels), which are further augmented after DPP4 inhibition (56), would be particularly sensitive to vagotomy. In support of this theory, the effects of intraportal administration of a DPP4i (improved glucose tolerance and increased insulin secretion) were abolished after vagotomy in rats (57). In our study, DPP4 inhibition, as expected, significantly increased insulin, C-peptide and ISR responses and decreased glucagon responses to the oral glucose load in the control subjects. However, the effect of the DPP4i was less clear in the vagotomised subjects, where there were no significant effects on insulin or glucagon secretion, in spite of higher levels of both intact GLP1 and GIP. A dose–response relationship for GLP1 (and GIP) would have been expected in the concentration range observed here (58). The lack of responses to these higher concentrations of GLP1 after vagotomy may therefore support the idea of at least partial transmission of GLP1's effect via vagal pathways.

Studies on patients after pancreas transplantation have suggested that pancreatic innervation may not be necessary for the incretin effect (59). However, comparison of the glucose data with that from a historical healthy control group (reported in that study) with identical incretin effect does suggest that the GIGD may have been reduced in the transplanted group. Dogs with a transplanted (and therefore denervated) pancreas have been reported to have reduced insulin responses (60) or hyperglycaemia (61) after oral, but not i.v., glucose, suggesting impaired incretin action. However, in another study, also in dogs, the incretin effect was preserved after pancreatic transplantation (62). In pigs, the incretin effect was unchanged after pancreas transplantation (63). The most likely explanation may be that both neural and endocrine mechanisms are operative. A similar situation exists regarding transmission of the food intake reducing effects of GLP1; there is good evidence that the effect of i.p. GLP1 in rats is transmitted via vagal efferents, but it is equally clear that i.v. GLP1 can inhibit food intake via a mechanism that is independent of the vagus nerves (16). In addition, it should be considered that the effect of the other incretin hormone, GIP, is thought to be independent of neural mechanisms (9, 13).

Oral glucose ingestion failed to suppress glucagon secretion in the vagotomised subjects, whereas the response was preserved after IIGI. A similar pattern has also been observed in patients with type 1 and type 2 diabetes, and it was suggested that gastrointestinal factors contribute to the impaired glucagon response to OGTT (21). These include GLP1, which strongly inhibits glucagon secretion (reviewed in (1, 47)), and GLP2 and GIP, both of which stimulate secretion (64, 65, 66). In this study, elevated concentrations of GIP coincided with higher glucagon concentrations and because GLP2 is co-secreted from the L cell in equimolar amounts with GLP1 (67), it would be expected that its levels were also increased. One study found lower suppression of glucagon secretion after oral than i.v. glucose in healthy humans subjects (68), but this was not found in several other studies (21, 69) and may be observed with very large oral glucose loads (69), not with 50 g as used here. Moreover, the control subjects in this study were able to suppress glucagon secretion equally irrespective of the route of glucose administration, illustrating that the glucagon response in the vagotomised subjects is an abnormal finding. Another possible explanation for the failed suppression of glucagon in the vagotomised subjects could be the accelerated gastric emptying, which clearly stimulated intestinal L cell secretion. Possibly, disturbed processing of proglucagon, the common precursor of glucagon and GLP1, could give rise to the release of immunoreactive glucagon (70). Interestingly, DPP4 inhibition, which increased levels of active GLP1 even further, suppressed glucagon secretion in controls and tended to normalise the glucagon response in the vagotomised subjects, without affecting gastric emptying, which would support a complex interaction of the various gut hormones on α cell secretion.

In conclusion, vagotomised subjects with pyloroplasty have altered glucose profiles after OGTT and a reduced GIGD. They exhibit a normal incretin effect, despite much higher levels of intact GLP1 and GIP, which could be consistent with the notion that the effect of GLP1 may be impaired after vagotomy. Furthermore, the vagotomised subjects had inappropriate glucagon responses despite the higher GLP1 levels and no effect of DPP4 inhibition on insulin and glucagon secretion. Therefore, these data support the suggestion that vagotomy may be associated with decreased effects of GLP1 on the endocrine pancreas, perhaps due to the loss of GLP1 signalling via vagal afferents. However, further studies aiming at eliminating the effects of the associated pyloroplasty in the vagotomised subjects are needed to clarify this.

## Supplementary data

This is linked to the online version of the paper at http://dx.doi.org/10.1530/EJE-13-0264.

## Author contribution statement

A Plamboeck contributed to the study design, researched data and wrote the manuscript. S Veedfald contributed with researched data and reviewed and edited the manuscript. S Meisner, A Wettergren, L B Svendsen and C Hovendal contributed with selection of the truncal vagotomised subjects and reviewed and edited the manuscript. C F Deacon and B Hartmann contributed to the discussion and reviewed and edited manuscript. F K Knop, T Vilsbøll and J J Holst contributed to the study design and reviewed and edited the manuscript. J J Holst also generated data. A Plamboeck is the guarantor of this work and, as such, had full access to all the data in the study and takes responsibility for the integrity of the data and the accuracy of the data analysis.

## Supplementary Material

Supplementary Table

## Figures and Tables

**Table 1:** Subjects' characteristics. Data are shown as means±s.e.m.

	**Vagotomised**	**Controls**
Age (years)	68±2	67±1
Sex (M/F)	16/0	10/0
BMI (kg/m^2^)	24±1	25±1
Waist:hip ratio	0.9±0	0.9±0
Systolic blood pressure (mmHg)	151±5	146±4
Diastolic blood pressure (mmHg)	87±3	96±10
Fasting plasma glucose (mM)	5.6±0.1	5.3±0.1
HbA1c (%)	5.9±0*	5.7±0.1
HOMA2–IR	1.3±0.3	1.1±0.2

HOMA, homeostasis model assessment; IR, insulin resistance. *P<0.02 vs control.

**Table 2:** Baseline values and tAUCs for PG and gastrointestinal hormones during OGTT and IIGI. Data are shown as means±s.e.m.

	**Vagotomised**	**Controls**	***P***
Glucose			
Mean baseline_OGTT_ (mM)	5.6±0.1	5.3±0.1	NS
Mean baseline_IIGI_ (mM)	5.6±0.1	5.4±0.1	NS
*P* (OGTT vs IIGI)	NS	NS	
tAUC_OGTT_ (mM×240 min)	1620±50	1380±40	<0.05
tAUC_IIGI_ (mM×240 min)	1735±52	1488±46	<0.05
*P* (OGTT vs IIGI)	<0.05	<0.05	
Total GLP1			
Mean baseline_OGTT_ (pM)	12±1	11±1	NS
Mean baseline_IIGI_ (pM)	10±1	10±1	NS
*P* (OGTT vs IIGI)	NS	NS	
tAUC_OGTT_ (pM×240 min)	7134±1079	3294±211	<0.05
tAUC_IIGI_ (pM×240 min)	2892±202	2186±225	<0.05
*P* (OGTT vs IIGI)	<0.05	<0.05	
Intact GLP1			
Mean baseline_OGTT_ (pM)	0.7±0.4	0.7±0.4	NS
Mean baseline_IIGI_ (pM)	0.5±0.2	0.6±0.4	NS
*P* (OGTT vs IIGI)	NS	NS	
tAUC_OGTT_ (pM×240 min)	1408±294	252±77	<0.05
tAUC_IIGI_ (pM×240 min)	161±64	52±30	NS
*P* (OGTT vs IIGI)	<0.05	<0.05	
Intact GIP			
Mean baseline_OGTT_ (pM)	21±1	20±1	NS
Mean baseline_IIGI_ (pM)	21±1	19±2	NS
*P* (OGTT vs IIGI)	NS	NS	
tAUC_OGTT_ (pM×240 min)	6643±337	5693±334	NS
tAUC_IIGI_ (pM×240 min)	4788±264	4090±244	NS
*P* (OGTT vs IIGI)	<0.05	<0.05	
Insulin			
Mean baseline_OGTT_ (pM)	59±12	49±7	NS
Mean baseline_IIGI_ (pM)	62±12	52±6	NS
*P* (OGTT vs IIGI)	NS	NS	
tAUC_OGTT_ (nM×240 min)	63±11	40±6	NS
tAUC_IIGI_ (nM×240 min)	31±7	20±4	<0.05
*P* (OGTT vs IIGI)	<0.05	<0.05	
C-peptide			
Mean baseline_OGTT_ (pM)	564±52	483±47	NS
Mean baseline_IIGI_ (pM)	587±59	512±48	NS
*P* (OGTT vs IIGI)	NS	NS	
tAUC_OGTT_ (nM×240 min)	425±32	313±28	<0.05
tAUC_IIGI_ (nM×240 min)	296±25	194.9±28	<0.05
*P* (OGTT vs IIGI)	<0.05	<0.05	
ISR			
tAUC_OGTT_ (pM/kg per min×240 min)	1325±98	902±87	<0.05
tAUC_IIGI_ (pM/kg per min×240 min)	889±63	544±75	<0.05
*P* (OGTT vs IIGI)	<0.05	<0.05	
Glucagon			
Mean baseline_OGTT_ (pM)	6.4±0.6	5.9±1.0	NS
Mean baseline_IIGI_ (pM)	6.6±0.6	7.1±1.1	NS
*P* (OGTT vs IIGI)	NS	NS	
tAUC_OGTT_ (pM×240 min)	1606±207	1184±147	NS
tAUC_IIGI_ (pM×240 min)	1196±134	1277±186	NS
*P* (OGTT vs IIGI)	<0.05	NS	

tAUC, total area under the curve; OGTT, 50 g-oral glucose tolerance test; IIGI, isoglycaemic i.v. glucose infusion; GLP1, glucagon-like peptide 1; GIP, glucose-dependent insulinotropic polypeptide; ISR, insulin secretion rate; NS, non-significant *P* value.

**Table 3:** tAUCs for PG and gastrointestinal hormones during absolute isoglycaemia for OGTT and IIGI. Data are shown as means±s.e.m.

	**Vagotomised**	**Controls**	***P***
Glucose			
tAUC_OGTT_ (mM×75 min)	728±33	577±26	<0.05
tAUC_IIGI_ (mM×75 min)	740±29	572±26	<0.05
*P* (OGTT vs IIGI)	NS	NS	
Total GLP1			
tAUC_OGTT_ (pM×75 min)	4902±1180	1378±120	<0.05
tAUC_IIGI_ (pM×75 min)	904±83	727±72	NS
*P* (OGTT vs IIGI)	<0.05	<0.05	
Intact GLP1			
tAUC_OGTT_ (pM×75 min)	780±143	133±43	<0.05
tAUC_IIGI_ (pM×75 min)	65±26	31±18	NS
*P* (OGTT vs IIGI)	<0.05	<0.05	
Intact GIP			
tAUC_OGTT_ (pM×75 min)	2887±182	2304±194	<0.05
tAUC_IIGI_ (pM×75 min)	1532±99	1269±76	NS
*P* (OGTT vs IIGI)	<0.05	<0.05	
Insulin			
tAUC_OGTT_ (nM×75 min)	43±5	26±4	<0.05
tAUC_IIGI_ (nM×75 min)	14±2	9±2	NS
*P* (OGTT vs IIGI)	<0.05	<0.05	
C-peptide			
tAUC_OGTT_ (nM×75 min)	185±13	129±14	<0.05
tAUC_IIGI_ (nM×75 min)	96±7	70±9	<0.05
*P* (OGTT vs IIGI)	<0.05	<0.05	
ISR			
tAUC_OGTT_ (pM/kg per min×75 min)	820±67	526±64	<0.05
tAUC_IIGI_ (pM/kg per min×75 min)	392±23	254±35	<0.05
*P* (OGTT vs IIGI)	<0.05	<0.05	
Glucagon			
tAUC_OGTT_ (pM×75 min)	555±70	327±50	<0.05
tAUC_IIGI_ (pM×75 min)	346±44	350±61	NS
*P* (OGTT vs IIGI)	<0.05	NS	

tAUC, total area under the curve; OGTT, 50 g-oral glucose tolerance test; IIGI, isoglycaemic i.v. glucose infusion; GLP1, glucagon-like peptide 1; GIP, glucose-dependent insulinotropic polypeptide; ISR, insulin secretion rate; NS, non-significant *P* value.

**Table 4:** Incretin effect (difference in insulin secretory responses during OGTT and IIGI). Data are shown as means±s.e.m. Incretin effect (100%×(AUC_OGTT_−AUC_IIGI_/AUC_OGTT_)) calculated from tAUC and iAUC during 75 and 240 min.

	**Vagotomised**	**Controls**	***P***
Insulin			
Incretin effect (tAUC_0–240 min_) (%)	50±4	54±4	NS
Incretin effect (iAUC_0–240 min_) (%)	62±4	72±4	NS
Incretin effect (tAUC_0–75 min_) (%)	64±4	64±3	NS
Incretin effect (iAUC_0–75 min_) (%)	72±3	78±3	NS
C-peptide			
Incretin effect (tAUC_0–240 min_) (%)	29±4	39±4	<0.05
Incretin effect (iAUC_0–240 min_) (%)	45±4	63±6	<0.05
Incretin effect (tAUC_0–75 min_) (%)	46±3	46±3	NS
Incretin effect (iAUC_0–75 min_) (%)	61±3	67±3	NS
ISR			
Incretin effect (tAUC_0–240 min_) (%)	31±4	40±4	NS
Incretin effect (tAUC_0–75 min_) (%)	49±3	51±4	NS

tAUC, total area under the curve; iAUC, incremental AUC; NS, non-significant *P* value.

**Table 5:** Effect of DPP4 inhibition: absolute difference in PG, gastrointestinal hormones and ISR between OGTT with and without DPP4 inhibition. Data are shown as means±s.e.m.

	**Vagotomised**	**Controls**	***P***
Glucose			
Diff (iAUC) (mM×240 min)	−5.6±27.6	−15.0±32.5	NS
Paracetamol			
Diff (time-to-peak) (min)	5.6±4.7	3.0±6.3	NS
Total GLP1			
Diff (iAUC) (pM×240 min)	−577±608	−421±166	NS
Intact GLP1			
Diff (iAUC) (pM×240 min)	1144±457	−7.4±124	NS
Intact GIP			
Diff (iAUC) (pM×240 min)	1679±488	2662±483	NS
Insulin			
Diff (iAUC) (pM×240 min)	10 406±6435	8824±3044	NS
C-peptide			
Diff (iAUC) (pM×240 min)	61 213±31 065	35 211±13 990	NS
ISR			
Diff (AUC) (pM×240 min)	248±116	132±56	NS
Glucagon			
Diff (iAUC) (pM×240 min)	−339±189	−478±139	NS

OGTT, 50 g-oral glucose tolerance test; DPP4i, dipeptidyl peptidase 4 inhibitor; Diff, absolute difference calculated from total area under the curve (tAUC) for ISR and from incremental AUC (iAUC) for PG and gastrointestinal hormones: (iAUC_OGTT__+__DPP4_−iAUC_OGTT_); GLP1, glucagon-like peptide 1; GIP, intact glucose-dependent insulinotropic polypeptide; ISR, insulin secretion rate; NS, non-significant *P* value.

**Table 6:** Baseline values and AUCs for PG and gastrointestinal hormones during OGTT with and without DPP4 inhibition. Data are shown as means±s.e.m.

	**Vagotomised**	**Controls**	***P***
Glucose			
Mean baseline_OGTT_ (mM)	5.6±01	5.3±0.1	<0.05
Mean baseline_OGTT__+__DDP4i_ (mM)	5.5±0.1	5.1±0.1	<0.05
*P* (OGTT vs OGTT+DPP4i)	<0.05	<0.05	
iAUC_OGTT_ (mM×240 min)	481±46	341±42	<0.05
iAUC_OGTT__+__DPP4i_ (mM×240 min)	475±45	326±35	<0.05
*P* (OGTT vs OGTT+DPP4i)	NS	NS	
Total GLP1			
Mean baseline_OGTT_ (pM)	11.9±1.1	10.7±1.0	NS
Mean baseline_OGTT__+__DDP4i_ (pM)	10.8±0.9	10.4±1.1	NS
*P* (OGTT vs OGTT+DPP4i)	NS	NS	
iAUC_OGTT_ (pM×240 min)	5118±1409	1133±146	<0.05
iAUC_OGTT__+__DPP4i_ (pM×240 min)	4542±1319	712±53	<0.05
*P* (OGTT vs OGTT+DPP4i)	NS	<0.05	
Intact GLP1			
Mean baseline_OGTT_ (pM)	0.7±0.4	0.7±0.4	NS
Mean baseline_OGTT__+__DDP4i_ (pM)	1.9±0.4	1.7±0.6	NS
*P* (OGTT vs OGTT+DPP4i)	<0.05	NS	
iAUC_OGTT_ (pM×240 min)	1245±263	85±85	<0.05
iAUC_OGTT__+__DPP4i_ (pM×240 min)	2389±664	78±64	<0.05
*P* (OGTT vs OGTT+DPP4i)	NS	NS	
Intact GIP			
Mean baseline_OGTT_ (pM)	20.9±1.1	19.8±0.9	NS
Mean baseline_OGTT__+__DDP4i_ (pM)	25.5±1.3	22.5±1.6	NS
*P* (OGTT vs OGTT+DPP4i)	<0.05	<0.05	
iAUC_OGTT_ (pM×240 min)	1645±263	917±217	NS
iAUC_OGTT__+__DPP4i_ (pM×240 min)	3310±509	3578±526	NS
*P* (OGTT vs OGTT+DPP4i)	<0.05	<0.05	
Insulin			
Mean baseline_OGTT_ (pM)	58.7±11.6	49.2±7.2	NS
Mean baseline_OGTT__+__DDP4i_ (pM)	61.0±10.1	56.2±10.5	NS
*P* (OGTT vs OGTT+DPP4i)	NS	NS	
iAUC_OGTT_ (nM×240 min)	49.5±7.9	29.6±4.6	<0.05
iAUC_OGTT__+__DPP4_ (nM×240 min)	59.9±11.8	38.4±7.1	NS
*P* (OGTT vs OGTT+DPP4i)	NS	<0.05	
C-peptide			
Mean baseline_OGTT_ (pM)	564±52	483±47	NS
Mean baseline_OGTT__+__DDP4i_ (pM)	613±54	589±62	NS
*P* (OGTT vs OGTT+DPP4i)	NS	NS	
iAUC_OGTT_ (nM×240 min)	289.2±24.4	198.5±19.4	<0.05
iAUC_OGTT__+__DPP4i_ (nM×240 min)	350.4±50.0	233.7±25.5	NS
*P* (OGTT vs OGTT+DPP4i)	NS	<0.05	
ISR			
AUC_OGTT_ (pM/kg per min×240 min)	1384±99	943±92	<0.05
AUC_OGTT__+__DPP4i_ (pM/kg per min×240 min)	1632±185	1076±113	<0.05
*P* (OGTT vs OGTT+DPP4i)	NS	<0.05	
Glucagon			
Mean baseline_OGTT_ (pM)	6.4±0.6	6.1±0.9	NS
Mean baseline_OGTT__+__DDP4i_ (pM)	7.6±0.8	7.9±1.4	NS
*P* (OGTT vs OGTT+DPP4i)	NS	<0.05	
iAUC_OGTT_ (pM×240 min)	102±121	−221±119	NS
iAUC_OGTT__+__DPP4i_ (pM×240 min)	−237±213	−698±240	NS
*P* (OGTT vs OGTT+DPP4i)	NS	<0.05	

iAUC, incremental area under the curve; OGTT, 50 g-oral glucose tolerance test; DPP4i, dipeptidyl peptidase 4 inhibitor; GLP1, glucagon-like peptide 1; GIP, glucose-dependent insulinotropic polypeptide; ISR, insulin secretion rate; NS, non-significant *P* value.
