# Supplementary material for: Characterisation of oral and i.v. glucose handling in truncally vagotomised subjects with pyloroplasty
Source: Eur J Endocrinol. 2013 May 21;169(2):187–201. doi: 10.1530/EJE-13-0264 (PMC3709640; doi:10.1530/EJE-13-0264)
Supplement: Supplementary Table [file supp_169_2_187__index.html]

Supplementary Table 

# Characterisation of oral and i.v. glucose handling in truncally vagotomised subjects with pyloroplasty

## 

**Files in this Data Supplement:**

- Supplementary Table 1 - (PDF 28 KB)
- Supplementary Table 2 - (PDF 35 KB)
- Supplementary Table 3 - (PDF 30 KB)
- Supplementary Table 4 - (PDF 29 KB)
- Supplementary Table 5 - (PDF 31 KB)
