# Supplementary material for: Characterisation of oral and i.v. glucose handling in truncally vagotomised subjects with pyloroplasty
Source: Eur J Endocrinol. 2013 May 21;169(2):187–201. doi: 10.1530/EJE-13-0264 (PMC3709640; doi:10.1530/EJE-13-0264)
Supplement: Supplementary Table [file supp_EJE-13-0264_Supplementary_table_1.pdf]

Table 1. Subjects characteristics, vagotomised subjects

	**Duodenal ulcer**	**Esophagus cancer**	***P***
**Age (years)**	71±2	65±2	NS
**Sex (M/F)**	7/0	9/0	NS
**BMI (kg/m^2)**	25±1	23±1	NS
**Waist:hip-ratio**	0.9±0	0.9±0	NS
**Systolic blood pressure (mmHg)**	140±9	160±5	NS
**Diastolic blood pressure (mmHg)**	85±4	89±3	NS
**Fasting plasma glucose (mM)**	5.8±0.3	5.4±0	NS
**HbA_{1c} (%)**	5.9±0	6.0±0.1	NS
**HOMA2-IR**	1.9±0.5	1.0±0.1	NS

Data are shown as means ± standard error of the mean (SEM). BMI, body mass index; HbA_{1c}, Hemoglobin A1c; HOMA, Homeostasis Model Assessment; IR, insulin resistance; NS, non-significant *P* value.
