# Supplementary material for: Characterisation of oral and i.v. glucose handling in truncally vagotomised subjects with pyloroplasty
Source: Eur J Endocrinol. 2013 May 21;169(2):187–201. doi: 10.1530/EJE-13-0264 (PMC3709640; doi:10.1530/EJE-13-0264)
Supplement: Supplementary Table [file supp_EJE-13-0264_Supplementary_table_2.pdf]

Table 2. Baseline values and tAUCs for PG and gastrointestinal hormones during OGTT and IIGI

	**Duodenal ulcer**	**Esophagus cancer**	***P***
**Glucose**			
Mean $baseline_{OGTT}$ (mM)	5.9±0.3	5.4±0.1	NS
Mean $baseline_{IIGI}$ (mM)	5.8±0.3	5.5±0.1	NS
$tAUC_{OGTT}$ (mM × 240 min)	1641±105	1604±43	NS
$tAUC_{IIGI}$ (mM × 240 min)	1755±95	1718±59	NS
**Total GLP-1**			
Mean $baseline_{OGTT}$ (pM)	14±2	10±1	NS
Mean $baseline_{IIGI}$ (pM)	12±1	9±1	NS
$tAUC_{OGTT}$ (pM × 240 min)	7126±824	7797±2622	NS
$tAUC_{IIGI}$ (pM × 240 min)	3183±321	2667±255	NS
**Intact GLP-1**			
Mean $baseline_{OGTT}$ (pM)	1.4±0.7	0.1±0.1	NS
Mean $baseline_{IIGI}$ (pM)	1.0±0.4	0.2±0.2	NS
$tAUC_{OGTT}$ (pM × 240 min)	1546±444	1301±412	NS
$tAUC_{IIGI}$ (pM × 240 min)	286±122	64±46	NS
**Intact GIP**			
Mean $baseline_{OGTT}$ (pM)	21±2	21±1	NS
Mean $baseline_{IIGI}$ (pM)	20±2	22±1	NS
$tAUC_{OGTT}$ (pM × 240 min)	6183±694	7001±254	NS
$tAUC_{IIGI}$ (pM × 240 min)	4568±480	4959±301	NS
**Insulin**			
Mean $baseline_{OGTT}$ (pM)	83±24	40±5	NS

Mean baseline$_{IIGI}$ (pM)	85±23	44±8	NS
tAUC$_{OGTT}$ (nM × 240 min)	80.4±21.7	50.1±6.3	NS
tAUC$_{IIGI}$ (nM × 240 min)	40.3±14.3	23.9±3.2	NS
**C-peptide**			
Mean baseline$_{OGTT}$ (pM)	717±75	446±41	<0.05
Mean baseline$_{IIGI}$ (pM)	730±103	476±42	<0.05
tAUC$_{OGTT}$ (nM × 240 min)	501.1±51.1	364.9±29.5	<0.05
tAUC$_{IIGI}$ (nM × 240 min)	354.2±49.8	250.0±10.7	<0.05
iAUC$_{OGTT}$ (nM × 240 min)	329.8±34.9	258.0±31.5	NS
iAUC$_{IIGI}$ (nM × 240 min)	180.0±31.4	136.8±11.5	NS
**ISR**			
tAUC$_{OGTT}$ (pM/kg/min × 240 min)	1464±117	1216±142	NS
tAUC$_{IIGI}$, (pM/kg/min × 240 min)	1016±121	790±45	NS
**Glucagon**			
Mean baseline$_{OGTT}$ (pM)	7±1	6±1	NS
Mean baseline$_{IIGI}$ (pM)	7±1	6±1	NS
tAUC$_{OGTT}$ (pM × 240 min)	1741±381	1500±236	NS
tAUC$_{IIGI}$ (pM × 240 min)	1302±242	1114±154	NS

Data are shown as means ± standard error of the mean (SEM). iAUC, incremental area under the curve; tAUC, total area under the curve; OGTT, 50g oral glucose tolerance test; IIGI, isoglycaemic intravenous glucose infusion; GLP-1, glucagon-like peptide-1; GIP, glucose-dependent insulinotropic polypeptide; ISR, insulin secretion rate; NS, non-significant *P* value.

There is a small difference in baseline of C-peptide between the vagotomised subjects treated for duodenal ulcer and esophagus cancer leading to a difference in the tAUC. There is no difference in iAUC for C-peptide between the vagotomised subjects treated for duodenal ulcer and esophagus cancer.
