# Supplementary material for: Characterisation of oral and i.v. glucose handling in truncally vagotomised subjects with pyloroplasty
Source: Eur J Endocrinol. 2013 May 21;169(2):187–201. doi: 10.1530/EJE-13-0264 (PMC3709640; doi:10.1530/EJE-13-0264)
Supplement: Supplementary Table [file supp_EJE-13-0264_Supplementary_table_3.pdf]

Table 3. tAUCs for PG and gastrointestinal hormones during absolute isoglycaemia for OGTT and IIGI

	**Duodenal ulcer**	**Esophagus cancer**	***P***
**Glucose**			
$tAUC_{OGTT}$ (mM × 75 min)	749±59	712±40	NS
$tAUC_{IIGI}$ (mM × 75 min)	749±53	734±35	NS
**Total GLP-1**			
$tAUC_{OGTT}$ (pM × 75 min)	4422±616	5275±2092	NS
$tAUC_{IIGI}$ (pM × 75 min)	1047±124	793±102	NS
**Intact GLP-1**			
$tAUC_{OGTT}$ (pM × 75 min)	781±204	778±210	NS
$tAUC_{IIGI}$ (pM × 75 min)	129±49	16±12	NS
**Intact GIP**			
$tAUC_{OGTT}$ (pM × 75 min)	2652±401	3069±72	NS
$tAUC_{IIGI}$ (pM × 75 min)	1485±195	1569±101	NS
**Insulin**			
$tAUC_{OGTT}$ (nM × 75 min)	52.6±10.4	37.9±13.8	NS
$tAUC_{IIGI}$ (nM × 75 min)	16.4±2.7	11.2±1.2	NS
**C-peptide**			
$tAUC_{OGTT}$ (nM × 75 min)	208.8±20.4	166.9±13.8	NS
$tAUC_{IIGI}$ (nM × 75 min)	114.8±12.4	81.8±3.8	NS
**ISR**			
$tAUC_{OGTT}$ (pM/kg/min × 75 min)	869±85	782±103	NS
$tAUC_{IIGI}$, (pM/kg/min × 75 min)	445±36	352±22	NS

**Glucagon**			
$tAUC_{OGTT}$ (pM × 75 min)	612±104	510±71	NS
$tAUC_{IIGI}$ (pM × 75 min)	377±79	322±52	NS

Data are shown as means ± standard error of the mean (SEM). tAUC, total area under the curve; OGTT, 50g oral glucose tolerance test; IIGI, isoglycaemic intravenous glucose infusion; GLP-1, glucagon-like peptide-1; GIP, glucose-dependent insulinotropic polypeptide; ISR, insulin secretion rate; NS, non-significant *P* value.
