# Supplementary material for: Characterisation of oral and i.v. glucose handling in truncally vagotomised subjects with pyloroplasty
Source: Eur J Endocrinol. 2013 May 21;169(2):187–201. doi: 10.1530/EJE-13-0264 (PMC3709640; doi:10.1530/EJE-13-0264)
Supplement: Supplementary Table [file supp_EJE-13-0264_Supplementary_table_4.pdf]

Table 4. Incretin effect (difference in insulin secretory responses during OGTT and IIGI)

	Duodenal ulcer	Esophagus cancer	*P*
**Insulin**			
Incretin effect ($tAUC_{0\text{-}240\ min}$), %	50.6±7.6	50.0±5.3	NS
Incretin effect ($iAUC_{0\text{-}240\ min}$), %	62.5±6.3	62.2±4.5	NS
Incretin effect ($tAUC_{0\text{-}75\ min}$), %	61.6±7.2	66.6±3.5	NS
Incretin effect ($iAUC_{0\text{-}75\ min}$), %	70.6±6.4	73.8±3.1	NS
**C-peptide**			
Incretin effect ($tAUC_{0\text{-}240\ min}$), %	29.8±6.1	29.0±4.4	NS
Incretin effect ($iAUC_{0\text{-}240\ min}$), %	46.1±6.3	43.7±5.6	NS
Incretin effect ($tAUC_{0\text{-}75\ min}$), %	42.8±6.6	49.1±3.3	NS
Incretin effect ($iAUC_{0\text{-}75\ min}$), %	58.1±5.7	63.4±3.3	NS
**ISR**			
Incretin effect, % ($tAUC_{0\text{-}240\ min}$)	30.7±5.9	31.5±4.5	NS
Incretin effect ($tAUC_{0\text{-}75\ min}$), %	46.2±6.1	51.9±3.6	NS

Data are shown as means ± standard error of the mean (SEM). Incretin effect [100% x (AUC_{OGTT} - AUC_{IIGI}/AUC_{OGTT})] calculated from total area under the curve (tAUC) and incremental area under the curve (iAUC) during 75 and 240 minutes. NS, non-significant *P* value.
