# Supplementary material for: Characterisation of oral and i.v. glucose handling in truncally vagotomised subjects with pyloroplasty
Source: Eur J Endocrinol. 2013 May 21;169(2):187–201. doi: 10.1530/EJE-13-0264 (PMC3709640; doi:10.1530/EJE-13-0264)
Supplement: Supplementary Table [file supp_EJE-13-0264_Supplementary_table_5.pdf]

Table 5. Baseline values and AUCs for PG and gastrointestinal hormones during OGTT with and without DPP-4 inhibition

	**Duodenal ulcer**	**Esophagus cancer**	***P***
**Glucose**			
Mean baseline$_{OGTT+DDP-4i}$ (mM)	5.6±0.3	5.4±0.1	NS
iAUC$_{OGTT}$ (mM × 240 min)	536±76	438±57	NS
iAUC$_{OGTT+DPP-4i}$ (mM × 240 min)	511±73	448±59	NS
**Total GLP-1**			
Mean baseline$_{OGTT+DDP-4i}$ (pM)	12±2	10±1	NS
iAUC$_{OGTT}$ (pM × 240 min)	4837±1107	5463±2423	NS
iAUC$_{OGTT+DPP-4i}$ (pM × 240 min)	4337±1557	3823±1991	NS
**Intact GLP-1**			
Mean baseline$_{OGTT+DDP-4i}$ (pM)	2±1	2±0	NS
iAUC$_{OGTT}$ (pM × 240 min)	1202±314	1279±416	NS
iAUC$_{OGTT+DPP-4i}$ (pM × 240 min)	3213±877	1748±951	NS
**Intact GIP**			
Mean baseline$_{OGTT+DDP-4i}$ (pM)	26±2	25±2	NS
iAUC$_{OGTT}$ (pM × 240 min)	1218±465	1977±271	NS
iAUC$_{OGTT+DPP-4i}$ (pM × 240 min)	3223±883	3378±637	NS
**Insulin**			
Mean baseline$_{OGTT+DDP-4i}$ (pM)	76.5±20.2	48.9±7.9	NS
iAUC$_{OGTT}$ (nM × 240 min)	60.9±15.9	40.5±6.1	NS
iAUC$_{OGTT+DPP-4}$, (nM × 240 min)	81.7±18.9	42.9±13.1	NS

**C-peptide**			
Mean baseline$_{OGTT+DDP-4i}$ (pM)	747±85	508±48	NS
iAUC$_{OGTT}$ (nM × 240 min)	324.4±34.9	257.0±31.5	NS
iAUC$_{OGTT+DPP-4i}$ (nM × 240 min)	428.2±51.2	289.9±63.6	NS
**ISR**			
AUC$_{OGTT}$ (pM/kg/min × 240 min)	1529±120	1271±144	NS
AUC$_{OGTT+DPP-4i}$ (pM/kg/min × 240 min)	1872±185	1445±290	NS
**Glucagon**			
Mean baseline$_{OGTT+DDP-4i}$ (pM)	8.4±1.5	7.0±0.7	NS
iAUC$_{OGTT}$ (pM × 240 min)	4.9±243	178±113	NS
iAUC$_{OGTT+DPP-4i}$ (pM × 240 min)	-161±405	-297±232	NS

Data are shown as means ± standard error of the mean (SEM). iAUC, incremental area under the curve; OGTT, 50g oral glucose tolerance test; DPP-4i, dipeptidyl peptidase 4 inhibitor; GLP-1, glucagon-like peptide-1; GIP, glucose-dependent insulinotropic polypeptide; ISR, insulin secretion rate; NS, non-significant *P* value.
